# Molecular and Clinical Characterization of the Variable Phenotype in Korean Families with Hearing Loss Associated with the Mitochondrial A1555G Mutation

**DOI:** 10.1371/journal.pone.0042463

**Published:** 2012-08-06

**Authors:** Jae Woong Bae, Dong-Bin Kim, Jae Young Choi, Hong-Joon Park, Jong Dae Lee, Dong Gu Hur, Seung-Hyun Bae, Da Jung Jung, Sang Heun Lee, Un-Kyung Kim, Kyu Yup Lee

**Affiliations:** 1 Department of Biology, College of Natural Sciences, Kyungpook National University, Daegu, South Korea; 2 Department of Otorhinolaryngology, Yonsei University College of Medicine, Seoul, South Korea; 3 Soree Ear Clinic, Seoul, South Korea; 4 Department of Otorhinolaryngology-Head and Neck Surgery, Soonchunhyang University College of Medicine, Bucheon, South Korea; 5 Department of Otorhinolaryngology-Head and Neck Surgery, Gyeongsang National University Hospital, Jinju, South Korea; 6 Department of Otolaryngology, College of Medicine, Kyungpook National University, Daegu, South Korea; Instituto de Investigación Hospital 12 de Octubre, Spain

## Abstract

Hearing loss, which is genetically heterogeneous, can be caused by mutations in the mitochondrial DNA (mtDNA). The A1555G mutation of the 12S ribosomal RNA (rRNA) gene in the mtDNA has been associated with both aminoglycoside-induced and non-syndromic hearing loss in many ethnic populations. Here, we report for the first time the clinical and genetic characterization of nine Korean pedigrees with aminoglycoside-induced and non-syndromic hearing loss. These Korean families carry in the A1555G mutation of 12S rRNA gene and exhibit variable penetrance and expressivity of hearing loss. Specifically, the penetrance of hearing loss in these families ranged between 28.6% and 75%, with an average of 60.8%. These results were higher than the 29.8% penetrance that was previously reported in a Chinese population but similar to the 65.4% and 54.1% penetrance observed in a large Arab-Israeli population and nineteen Spanish pedigrees, respectively. The mutational analysis of the complete mtDNA genome in these families showed that the haplogroups of the Korean population, which belongs to the eastern Asian population, were similar to those of the Chinese population but different from the Spanish population, which belongs to the European-Caucasian population. The mtDNA variants that may act as modifier factors were also found to be similar to the Chinese population. Although the mtDNA haplogroups and variants were similar to the eastern Asian population, we did find some differing phenotypes, although some subjects had the same variants. This result suggests that both the ethnic background and environmental factors lead to a variable phenotype of the A1555G mutation.

## Introduction

Hearing loss is the most common sensorineural disorder in humans, affecting one in 1000 newborns and 10% and 50% of people aged 65 and 80 years or older, respectively [1]. It is genetically heterogeneous and can be caused by mitochondrial DNA (mtDNA) mutations [2]. MtDNA mutations have been reported in both non-syndromic and syndromic hearing loss such as Kearns-Sayre Syndrome (KSS) [3], myoclonic epilepsy and ragged red fibers (MERRF) [4], mitochondrial encephalopathy, lactic acidosis and stroke-like episodes (MELAS) [5], maternally inherited diabetes and deafness (MIDD) [6] and are associated with presbycusis [7], [8].

The A1555G mutation of the 12S ribosomal RNA (rRNA) gene in the mtDNA is associated with both aminoglycoside-induced and non-syndromic hearing loss in many ethnic populations [9], [10], [11], [12]. Transition of A to G results in an additional G–C pair in the 12S rRNA gene, which has been predicted to encode an aminoglycoside binding based on sequence similarity to the bacterium *Escherichia coli* [13], [14]. In addition, sporadic aminoglycoside-induced hearing loss has been reported [15], and even without the use of antibiotics, non-syndromic hearing loss also occurs in ethnic families. Families with hearing loss caused by the A1555G mutation in the 12S rRNA gene have variable phenotypes, including varying severity, age of onset, and penetrance [2], [9],[16],[17],[18]. Penetrance appears to differ with the use of aminoglycosides, even within the same pedigree [19]. The variable phenotypes of hearing loss in persons carrying the A1555G mutation are difficult to explain because it is a single point mutation. Therefore, additional modifying factors, including the mtDNA haplogroup, nuclear DNA or mtDNA variations and aminoglycoside antibiotics, have been proposed to be associated with the variable phenotypic expression [18], [20], [21]. A nuclear modifier gene, tRNA 5-methylaminomethyl-2-thiouridylate methyltransferase (*TRMU)*, has been identified, and this gene encodes a highly conserved mitochondrial protein related to transfer RNA (tRNA) modification [22]. The mtDNA variations that have been shown to influence the variable phenotype of hearing loss associated with the A1555G mutation are tRNA^Lys^ T5802C [18] and G5821A [19], tRNA^Ser(UCN)^ G7444A [23], tRNA^Arg^ T10454C [24], tRNA^Glu^ A14693G [24], [25], tRNA^Thr^ T15908C [24] and G15927A [18], and T12338C [18] in the ND5 gene. Ten eastern Asian haplogroups, including A, B, C, D, F, G, M, N, R and Y, have been detected in Chinese pedigrees carrying the A1555G mutation [26]. Seven European haplogroups have been detected in Spanish pedigrees, including H, I, J, K, T, U and K [27]. Notably, the frequency of the A1555G mutation is much higher in haplogroups D and H in the Chinese and Spanish populations, respectively, than the other haplogroups [26], [27]. However, the A1555G mutation has been detected in all of the haplogroups, suggesting that the A1555G mutation in the mtDNA occurred sporadically and persisted over generations. Chinese pedigrees carrying the haplogroups C, Y and F2 have been shown to have higher penetrance than the pedigrees carrying the other haplogroups [26].

In the present study, we investigated the association of modifier factors and variable phenotypes of hearing loss in Korean pedigrees carrying the A1555G mutation. We performed clinical, molecular, and genetic characterizations of the pedigrees, including a sequence analysis of the complete mtDNA genome.

## Subjects and Methods

### Subjects and Audiological Evaluation

A total of 281 unrelated Korean subjects with non-syndromic hearing loss participated for the mtDNA A1555G mutation screening. All the subjects were subjected to appropriate audiological examinations, including pure-tone audiometry (PTA) and/or auditory brainstem response (ABR). The average of pure-tone audiometry (PTA) was calculated from the average of the audiometric thresholds at 500, 1000, 2000, and 3000 Hz. The severity of hearing loss was classified as follows: normal <26 decibels (dB), mild; 26–40 dB, moderate; 41–70 dB, severe; 71–90 dB, and profound; >90 dB. The subjects with the mtDNA A1555G mutation were subjected to a comprehensive history interview and physical examination to identify other symptoms and their history of aminoglycoside use. All subjects provided written informed consent according to the protocol approved by the Ethics Committee of Kyungpook National University Hospital prior to the study.

### Mutation and Haplogroup Analysis of the mtDNA Genome

Genomic DNA was extracted from the peripheral blood of subjects using the Qiagen Flexigene DNA Extraction Kit (Qiagen, Hilden, Germany). PCR amplification of the mitochondrial 12S rRNA gene was performed using the following primers: forward, 5′- tggctttaacatatctgaacaca-3′, and reverse, 5′-ctcctaagtgtaagttgggtgct-3′. For the identification of the A1555G mutation, the PCR products were analyzed using PCR-RFLP with *Bsm*AI (New England Biolabs, Ipswich, MA, USA) [28]. To confirm the A1555G mutation, the PCR products were purified with the Exo-SAP enzyme (USB, Cleveland, OH, USA) and analyzed through direct sequencing on an ABI 3130 Genetic Analyzer (Applied Biosystems Corps., Foster City, CA, USA) using the Big-Dye Terminator Cycle Sequencing Kit (Applied Biosystems Corps., Foster City, CA, USA).

The complete mtDNA sequences of the subjects with the A1555G mutation were amplified (Table S1), purified with the Exo-SAP enzyme (USB, Cleveland, OH, USA), and analyzed through direct sequencing. All of the mtDNA sequences were compared with the updated consensus Cambridge Sequence (GenBank accession number: NC_012920).

### Mutation Analysis of the *GJB2* and *TRMU* Genes

PCR amplification of the exon 2 of the *GJB2* gene was performed using the following primers: forward, 5′-gcattcgtcttttccagagc-3′, and reverse, 5′-cctcatccctctcatgctgt-3′. The PCR products were purified with the Exo-SAP enzyme (USB, Cleveland, OH, USA) and analyzed through direct sequencing. The results were compared with the sequence of the wild-type *GJB2* gene (GenBank accession number: NM_004004) to identify mutations. For the identification of the *TRMU* gene mutation G28T (A10S), the PCR amplification of exon 1 of the *TRMU* gene was performed using a previously reported primer, and the PCR products were analyzed using PCR-RFLP with *Bsp*1286I (New England Biolabs, Ipswich, MA, USA) [22]. The digested products were analyzed on a 2% agarose gel.

## Results

### Mutational Screening of the 12S rRNA Gene in Korean Subjects with Non-syndromic Hearing Loss

**Figure 1:**
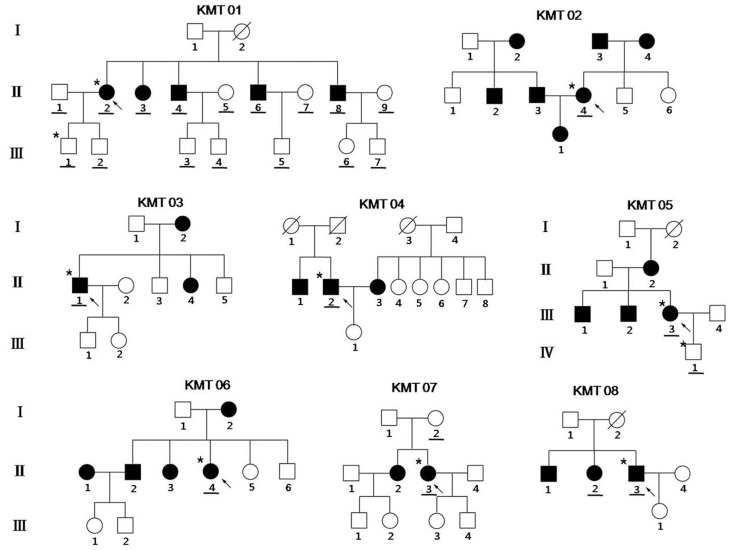
Eight Korean pedigrees presenting with nonsyndromic hearing loss were carrying the A1555G mtDNA mutation. Hearing-impaired individuals are indicated by filled symbols. Arrows denote probands. Subjects used for whole mtDNA sequence analysis are indicated by asterisks. Subjects used for the A1555G mutation screening are underlined.

We performed a sequence analysis to identify the A1555G mutation in 281 Korean unrelated subjects with non-syndromic hearing loss, excluding those subjects with complete autosomal recessive inheritance patterns. First, the genomic DNA of each subject was amplified using the appropriate primers, and these products were digested using *Bsm*AI and analyzed on a 2% agarose gel. Nine of the subjects had the A1555G mutation, which was further confirmed using PCR and subsequent DNA sequence analysis. Next, we performed mutational screening for the A1555G mutation in the available matrilineal relatives of those subjects except for KMT09 family who was not available for pedigree data. The A1555G mutation was detected in all matrilineal relatives. The penetrance of hearing loss (affected matrilineal relatives/total matrilineal relatives) of the eight pedigrees ranged from 28.6% to 75%, with an average of 60.8% (Fig. 1) [17], [19], [23], [24].

### Clinical and Genetic Evaluation of the Nine Korean Pedigrees

We obtained a comprehensive history and performed physical and audiological examinations to identify any syndromic symptoms, the history of aminoglycoside use and genetic factors in all of the available subjects of the nine pedigrees carrying the A1555G mutation (Table 1). The results showed that the probands and members of the nine pedigrees showed no other clinical abnormalities, including diabetes, muscular diseases, visual dysfunction, and neurological disorders.

**Table 1:** Summary of clinical features and molecular data for nine patients carrying the A1555G mutation.

Patient no	Gender	Age at test	Age of onset (Pre- or Postlingual)	Audiometric configuration	Exposure to aminoglycosides	PTA	(dB)*	Degree of Hearing loss	mtDNA haplogroup
						Right ear	Left ear		
KMT 01	F	51	–	Slope	No	90	93	Severe	D4b1b1a
KMT 02	F	26	Congenital (prelingual)	Slope	No	101	94	Severe	D4a
KMT 03	M	49	–	Slope	–	105	96	Profound	M7a1a
KMT 04	M	57	Childhood (postlingual)	–	–	111	112	Profound	D5a2a
KMT 05	F	41	R: Late childhood, L: 38 (postlingual)	Slope	No	104	101	Profound	D5b1b1
KMT 06	F	47	Late childhood (postlingual)	Slope	No	97	102	Profound	G1a1a
KMT 07	F	45	Childhood (postlingual)	Slope	Yes	73	67	Moderate	D4a
KMT 08	M	50	Congenital (prelingual)	Slope	No	103	117	Profound	M11b
KMT 09	F	67	Childhood (postlingual)	Slope	Yes	102	95	Profound	D4

* PTA, pure-tone audiometry; dB, decibels.

**Figure 2:**
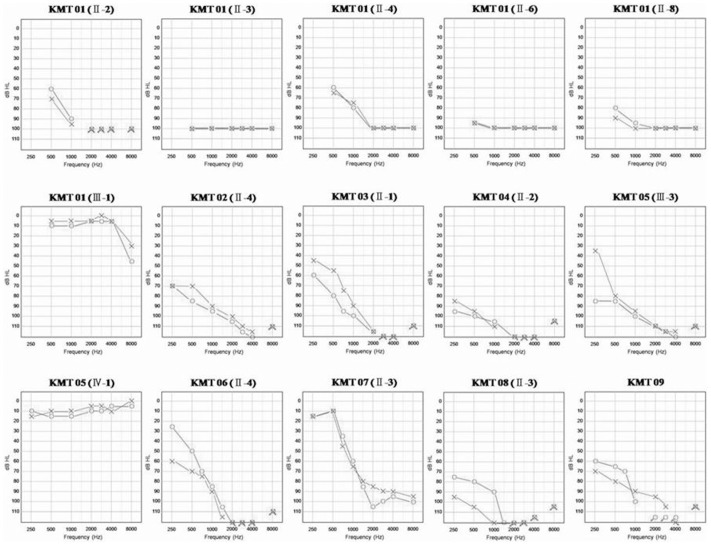
Air audiograms for pure tone audiometry (PTA) of the available subjects with the A1555G mutation. Arrows indicate no responses; Symbols, (X) left ear (O) right ear; dB HL, decibel hearing level.

The probands of each pedigree exhibited hearing loss ranging from moderate to profound, with a slope-shaped pattern of audiological evaluation (Fig. 2). Only the probands of the KMT 07 and KMT 09 families had a history of exposure to aminoglycosides. For the age at onset, the probands of the KMT 02 and KMT 08 families showed prelingual hearing loss, and those of the KMT 04, KMT 07 and KMT 09 families had postlingual hearing loss (Table 1).

The examination of the clinical information of the KMT 01 pedigree (Fig. 1 and 2 and Table 1) revealed that subjects II-3 and II-6 of the proband’s siblings (II-3, II-4, II-6, and II-8) had profound hearing loss with a flat-shaped pattern, and subjects II-4 and II-8 had severe hearing loss with a slope-shaped pattern. However, the proband’s son, subject III-1, had normal hearing, with only high-frequency hearing loss (30 dB in the right ear and 40 dB in the left ear at 8000 Hz). Husband II-3 of proband II-4 in the KMT 02 family had acquired hearing loss, and the proband’s daughter III-1 had congenital hearing loss. The reason for the hearing loss of subject III-1 was not known. Proband III-3 in the KMT 05 family had prelingual hearing loss in the right ear, but the hearing in the left ear became poor at 38 years of age. The onset of hearing loss in her siblings (III-1 and III-2) occurred during childhood and adulthood, respectively. In generations II and III, the penetrance of the A1555G mutation was 100%, but subject IV-1 had normal hearing. The proband’s siblings II-1 and II-2 of the KMT 08 family all had hearing loss, and the hearing loss of proband II-3 and subject II-2 was congenital.

### Haplogroup Analysis of the mtDNA Genome

To determine whether mtDNA variants or haplogroups modulated the variable phenotype of hearing loss in patients carrying the A1555G mutation, we performed a complete mtDNA sequence analysis of the probands and matrilineal members of the nine pedigrees. As shown in Table 2, the members of each pedigree had distinct mtDNA polymorphisms. Of the known nucleotide variations in the complete mtDNA sequence, we found thirty-five in the D-loop, six in the tRNA gene, nine in the 12S rRNA gene, and seven in the 16S rRNA gene. There were 83 variants in the protein-coding genes, including 56 silent variants and 27 missense variants. One novel variant of the 27 amino acid substitution variants was G3496A in the ND1 gene, which resulted in the substitution of the amino acid alanine with threonine (Table 2).

**Table 2:** mtDNA variants in nine Korean families with hearing loss.

			Conservation	KMT 01	KMT 01	KMT 02	KMT 03	KMT 04	KMT 05	KMT 05	KMT 06	KMT 07	KMT 07	KMT 08	KMT 09	
Gene	Position	Replacement	(H/B/M/X)^a^	II-2	III-1	II-4	II-1	II-2	III-3	IV-1	II-4	II-3	I-2	II-3		Previously reported^b^
*D-loop*	44	C>CC						CC								Yes
	73	A>G		G	G	G	G	G	G	G	G	G	G	G	G	Yes
	146	T>C												C		Yes
	150	C>T						T	T	T	T					Yes
	152	T>C				C						C	C		C	Yes
	183	A>G							G	G						Yes
	215	A>G												G		Yes
	228	G>A										A	A			Yes
	263	A>G		G	G	G	G	G	G	G	G	G	G	G	G	Yes
	310	T>CTC		CTC	CTC	CCTC	CTC	CTC	CCTC	CCTC	TC	CTC	CTC	CTC	CTC	Yes
	318	T>C												C		Yes
	326	A>G												G		Yes
	431	C>T		T	T											Yes
	456	C>T							T	T						Yes
	489	T>C		C	C	C	C	C	C	C	C	C	C	C	C	Yes
	515	Del AC		Del AC	Del AC		Del AC	Del AC								Yes
	681	T>C							C	C						Yes
	16092	T>C						C								Yes
	16129	G>A				A						A	A			Yes
	16164	A>G						G								Yes
	16182	A>C						C	C	C						Yes
	16183	A>C						C	C	C						Yes
	16189	T>C						C	C	C						Yes
	16209	T>C					C									Yes
	16223	C>T		T	T	T	T	T	T	T	T	T	T	T	T	Yes
	16266	C>T						T								Yes
	16287	C>T		T	T											Yes
	16319	G>A		A	A											Yes
	16324	T>C					C									Yes
	16325	T>C									C					Yes
	16357	T>C							C	C						Yes
	16362	T>C		C	C	C		C	C	C	C	C	C		C	Yes
	16399	A>G		G	G											Yes
	16497	A>G												G		Yes
	16519	T>C				C			C	C	C			C		Yes
*12s rRNA*	709	G>A	G/G/A/−								A					Yes
	750	A>G	A/G/G/−	G	G	G	G	G	G	G	G	G	G	G	G	Yes
	752	C>T						T								Yes
	1048	C>T							T	T						Yes
	1095	T>C												C		Yes
	1107	T>C	T/CT/T					C	C	C						Yes
	1310	C>T						T								Yes
	1438	A>G	A/A/A/G	G	G	G	G		G	G	G	G	G	G	G	Yes
	1462	G>A				A										Yes
	1555	A>G	A/A/A/A	G	G	G	G	G	G	G	G	G	G	G	G	Yes
*16s rRNA*	1811	A>G				G										Yes
	2626	T>C					C									Yes
	2706	A>G	A/G/A/A	G	G	G	G	G	G	G	G	G	G	G	G	Yes
	2772	C>T					T									Yes
	3010	G>A		A	A	A						A	A		A	Yes
	3107	Del C		Del C	Del C	Del C	Del C	Del C	Del C	Del C	Del C	Del C	Del C	Del C	Del C	Yes
	3206	C>T				T						T	T			Yes
*ND1*	3316	G>A (Ala to Thr)						A								Yes
	3496	G>A (Ala to Thr)	A/A/L/S						A	A						No
	3759	A>G							G	G						Yes
*TQ*	4386	T>C					C									Yes
*ND2*	4769	A>G		G	G	G	G	G	G	G	G	G	G	G	G	Yes
	4793	A>G									G					Yes
	4833	A>G (Thr to Ala)									G					Yes
	4859	T>C		C	C											Yes
	4883	C>T		T	T	T		T	T	T		T	T		T	Yes
	4958	A>G					G									Yes
	5108	T>C									C					Yes
	5147	G>A							A	A						Yes
	5153	A>G							G	G						Yes
	5178	C>A (Leu to Met)	L/T/T/T	A	A	A		A	A	A		A	A		A	Yes
	5276	A>G										G	G			Yes
	5301	A>G (Ile to Val)	I/I/M/L					G	G	G						Yes
*TC*	5802	T>C	T/T/T/C						C	C						Yes
*NC5*	5895	C>CC					CC								CC	Yes
*CO1*	6253	T>C (Met to Thr)							C	C						Yes
	6410	C>T		T	T											Yes
	6455	C>T					T									Yes
	6531	C>T												T		Yes
	6551	C>T	N/−/N/−									T	T			Yes
	6689	C>T		T	T											Yes
	7028	C>T		T	T	T	T	T	T	T	T	T	T	T	T	Yes
	7403	A>G							G	G						Yes
	7444	G>A (Ter to Lys)				A										Yes
*CO2*	7642	G>A												A		Yes
	7867	C>T									T					Yes
	8020	G>A		A	A											Yes
	8071	A>G						G								Yes
	8108	A>G (Ile to Val)	I/I/I/I											G		Yes
	8176	T>C						C								Yes
	8200	T>C									C					Yes
*TK*	8308	A>G					G									Yes
*ATP8*	8414	C>T (Leu to Phe)	L/F/M/W	T	T	T						T	T		T	Yes
	8473	T>C				C						C	C			Yes
*ATP6*	8701	A>G (Thr to Ala)	T/S/L/Q	G	G	G	G	G	G	G	G	G	G	G	G	Yes
	8860	A>G (Thr to Ala)	T/A/A/T	G	G	G	G	G	G	G	G	G	G	G	G	Yes
	9180	A>G						G	G	G						Yes
*CO3*	9254	A>G									G					Yes
	9531	A>G (Thr to Ala)													G	Yes
	9540	T>C		C	C	C	C	C	C	C	C	C	C	C	C	Yes
	9824	T>C					C									Yes
	9948	G>A (Val to Ile)							A	A						Yes
	9950	T>C												C		Yes
*ND3*	10084	T>C (Ile to Thr)		C	C											Yes
	10181	C>T		T	T											Yes
	10397	A>G						G	G	G						Yes
	10398	A>G (Thr to Ala)	T/T/T/A	G	G	G	G	G	G	G	G	G	G	G	G	Yes
	10400	C>T		T	T	T	T	T	T	T	T	T	T	T	T	Yes
*TR*	10438	A>G	A/A/A/G											G		Yes
*ND4L*	10685	G>A												A		Yes
*ND4*	10867	C>T	I/F/L/L	T	T											Yes
	10873	T>C		C	C	C	C	C	C	C	C	C	C	C	C	Yes
	11017	T>C					C									Yes
	11084	A>G (Thr to Ala)					G									Yes
	11719	G>A		A	A	A	A	A	A	A	A	A	A	A	A	Yes
	11914	G>A									A					Yes
	11944	T>C						C								Yes
	11969	G>A (Ala to Thr)												A		Yes
	12026	A>G (Ile to Val)						G								Yes
	12100	A>G	L/L/L/L												G	Yes
*TH*	12172	A>G		G	G											Yes
*ND5*	12705	C>T		T	T	T	T	T	T	T	T	T	T	T	T	Yes
	12771	G>A					A									Yes
	13074	A>G												G		Yes
	13278	A>G						G								Yes
	13528	A>G (Thr to Ala)		G	G											Yes
	13890	C>T												T		Yes
	13928	G>T (Ser to Ile)	S/T/S/T											T		Yes
*ND6*	14364	G>A					A									Yes
	14569	G>A									A					Yes
	14668	C>T		T	T	T						T	T		T	Yes
*CYB*	14766	C>T (Thr to Ile)	T/S/I/S	T	T	T	T	T	T	T	T	T	T	T	T	Yes
	14783	T>C		C	C	C	C	C	C	C	C	C	C	C	C	Yes
	14790	A>G (Asn to Ser)												G		Yes
	14979	T>C (Ile to Thr)	I/I/L/L			C						C	C			Yes
	15043	G>A		A	A	A	A	A	A	A	A	A	A	A	A	Yes
	15265	C>T												T		Yes
	15301	G>A		A	A	A	A	A	A	A	A	A	A	A	A	Yes
	15323	G>A (Ala to Thr)									A					Yes
	15326	A>G (Thr to Ala)	T/M/I/I	G	G	G	G	G	G	G	G	G	G	G	G	Yes
	15440	T>C		C	C											Yes
	15497	G>A (Gly to Ser)									A					Yes
	15724	A>G							G	G						Yes
	15748	T>C							C	C						Yes
	15860	A>G (Ile to Val)									G					Yes
*TT*	15951	A>G		G	G											Yes

a Conservation of amino acids for polypeptides or nucleotides for RNAs in human (H), bovine (B), mouse (M), and *Xenopus laevis* (X).

b See the online mitochondrial genome database http://www.mitomap.org.

Three major haplogroups, D, M and G, were detected in the mtDNA haplogroup analysis of the nine pedigrees. Haplogroups D4, D4b1b1a, D5a2a, D5b1b1, G1a1a, M7a1a and M11b were each found in seven pedigrees, and haplogroup D4a was found in two pedigrees. Haplogroup D was found in six pedigrees and was the most prevalent haplogroup in the nine pedigrees (Table 1).

### Mutational Analysis of the *GJB2* and *TRMU* Genes

To assess the role of the *GJB2* gene in the variable phenotype or existence of the mutation in the patients carrying the A1555G mutation, we performed a sequence analysis of the *GJB2* gene in all of the subjects with the A1555G mutation. None of the subjects had mutations in the *GJB2* gene. Additionally, the A10S mutation of the *TRMU* gene has been reported to be a modifier gene in hearing loss with the A1555G mutation. We also analyzed the DNA of the subjects using PCR-RFLP, and the A10S mutation was not detected in any of the subjects (data not shown).

## Discussion

The present study was performed in Korean subjects with non-syndromic clinically variable hearing loss carrying the A1555G mutation of the 12S rRNA gene in the mtDNA. To explain these variable phenotypes, we searched for mtDNA variants that acted as modifying factors of the variable phenotypes using complete mtDNA sequence analysis. First, the homoplasmic A1555G mutation of the 12S rRNA gene was detected in nine of the 281 unrelated subjects with non-syndromic hearing loss. Their pedigrees were characterized for clinical, genetic and molecular characteristics. The Korean pedigrees with hearing loss presented with wide penetrance and expressivity. The penetrance of the eight pedigrees (excluding the pedigree for KMT 09) ranged from 28.6% to 75%, with an average of 60.8%. These results were higher than the 29.5% penetrance observed in the previously reported Chinese population [26] but similar to the 65.4% and 54.1% penetrance of a large Arab-Israeli pedigree and nineteen Spanish pedigrees, respectively [9], [29]. This result suggested that the penetrance of hearing loss with the A1555G mutation was variable even within the same eastern population and appeared to differ among ethnic groups.

Mitochondrial haplogroups have been reported to be associated with diseases, including blindness [30], ageing [31], male infertility[32], Alzheimer’s [33], and diabetes [34]. In addition, mtDNA haplogroups have been shown to alter the phenotypic expression of syndromic and non-syndromic hearing loss. Lu et al. (2010) identified ten haplogroups in 69 pedigrees with hearing loss carrying the A1555G mutation: A, B, C, D, F, G, M, N, R and Y. Haplogroup D was found at a higher frequency in the hearing loss pedigrees than in 93 controls. In contrast, haplogroups A and M were found at lower frequencies in the hearing loss pedigrees than in the controls [26]. The mtDNA haplogroup analysis of the Spanish pedigrees revealed the following haplogroups: H, I, J, K, T, U, V and L [27], [35]. These haplogroups did not overlap with the haplogroups of the eastern Asian population. In the study of the Spanish pedigrees, 45.1% of individuals in the control group and 76% of the individuals in the hearing loss group were of haplogroup H, revealing a significantly higher percentage of this haplogroup in the hearing loss group [27]. In the present study, three major haplogroups, D, M and G, were detected in the nine pedigrees. Haplogroups D4, D4b1b1a, D5a2a, D5b1b1, G1a1a, M7a1a and M11b were each present in seven pedigrees, and haplogroup D4a was present in two pedigrees. A study analyzing the mtDNA haplogroups of 593 Koreans showed the following haplogroups: 4.9% haplogroup D4 and D4a, 2% D4b1, 2.2% D5a2, 2.7% G1a1, 1.3% M7a1 and 0.8% M11 [36]. Mitochondrial DNA haplogroups are restricted among ethnic populations. Haplogroup D in the eastern Asian population and haplogroup H in the Europe-Caucasian population are associated with hearing loss with the A1555G mutation [26], [27]. In the present study, haplogroup D was the most represented, similar to that found for the Chinese pedigrees. However, more pedigrees may be needed to estimate the association between an mtDNA haplogroup and hearing loss due to the mtDNA mutation.

Nuclear modifier genes have been reported to influence the variable phenotype of hearing loss with the A1555G mutation [22]. The mutant allele of the *MTO2* gene that encode mitochondrial proteins in yeast *S. cerevisiae* manifests a respiratory-deficient phenotype only when coupled with the paromomycin-resistance mitochondrial 15S rRNA 1409 C to T mutation [37]. This mutation corresponds to the human 12S rRNA 1494 C to T mutation. The *MTO2* gene is evolutionarily conserved and display sequence similarity to the human *TRMU* gene. Indeed, the missense mutation c.G28T (p.A10S) of the *TRMU* gene has been reported in hearing loss patients with the A1555G mutation in some ethnic populations [22]. However, the p.A10S mutation of the *TRMU* gene was not detected in the nine Korean pedigrees with hearing loss in this study.

Mitochondrial DNA variations have also been reported to influence the variable phenotype of mitochondrial disease, including the variable phenotype of hearing loss patients carrying the A1555G mutation. For example, the following mitochondrial tRNA variants may contribute to the phenotype: tRNA^Thr^ G15927A [18], tRNA^Cys^ T5802C [18], tRNA^Arg^ T10454C [24], tRNA^Ser(AGY)^ C12224T [26], tRNA^Cys^ G5821A [19], tRNA^Glu^ A14693G [24], tRNA^Thr^ T15908C [24], T12338C [18] of ND5, G7444A [23] of tRNA^Ser(UCN)^/CO1 and G11696A [26] of the ND4 gene. These mtDNA variants have been suggested to have significant effects on the penetrance and expressivity of hearing loss with the A1555G mutation. In this study, one novel mtDNA variant, G3496A of theND1 gene, was identified. Additionally, two variants of known modifier factors, tRNA^Cys^ T5802C and G7444A of the tRNA^Ser(UCN)^/CO1 gene, were identified. The G3496A variant, which causes a substitution of alanine to threonine at position 64 (p.A64T) of the ND1 gene, was analyzed for protein biochemical changes using the PolyPhen 2 (http://genetics.bwh.harvard.edu/pph2/), SNPs&GO (http://snps-and-go.biocomp.unibo.it/snps-and-go/) and Panther (http://www.pantherdb.org/tools/csnpScoreForm.jsp) programs. This substitution appears to have no association with the variable phenotype because all of the in silico tools predicted that this substitution is a benign polymorphism. The tRNA^Cys^ T5802C variant, however, has been reported to alter the structure of tRNAs and lead to a defect in tRNA metabolism [18]. Another variant, G7444A of the tRNA^Ser(UCN)^/CO1 gene, was not sufficient to produce a clinical phenotype [38]. Therefore, additional modifier factors, including nuclear backgrounds, environmental factors, and mitochondrial haplogroups, must alter the phenotypic manifestation. The variant G7444A of the tRNA^Ser(UCN)^/CO1 gene has been detected in several haplogroups, including C4a, B4 and D4a [26], [38], and was found in haplogroup D4a in this study. This result indicates that this variant was sporadic, similarly to the A1555G mutation. Additional studies are necessary to determine whether this variant affects the variable phenotype or is a simple polymorphism.

This study is the first to perform complete mtDNA sequencing to identify mtDNA haplogroups or variants in Korean pedigrees with non-syndromic hearing loss carrying the A1555G mutation. The haplogroups in the Korean population of the eastern Asian population are similar to those of the Chinese population but differ from the haplogroups of the Spanish populations of the Europe-Caucasian population. The mtDNA variants as modifier factors were also found to be similar to those of the Chinese population. The mtDNA haplogroups and variants are similar to the eastern Asian population but appear to have different phenotypes, although some subjects had the same variants [39], [40]. These results suggest that both the ethnic population and environmental factors lead to the variable phenotype of the A1555G mutation. However, this observation requires further pedigree and clinical evaluations to fully elucidate the mechanisms of the phenotypic manifestation of the A1555G mutation.

## Supporting Information

Table S1 **Primer sequences used for whole mtDNA genome analysis.** Bold sequences denote primers using PCR. Sequences of the rest are used for internal sequence primers.
(DOC)
