# Supplementary material for: Molecular and Clinical Characterization of the Variable Phenotype in Korean Families with Hearing Loss Associated with the Mitochondrial A1555G Mutation
Source: PLoS One. 2012 Aug 6;7(8):e42463. doi: 10.1371/journal.pone.0042463 (PMC3412860; doi:10.1371/journal.pone.0042463)
Supplement: Table S1 — Primer sequences used for whole mtDNA genome analysis. Bold sequences denote primers using PCR. Sequences of the rest are used for internal sequence primers. (DOC) [file pone.0042463.s001.doc]

**Supplementary Table 1.** Primer sequences used for whole mtDNA genome analysis

| No. | Position | Forward (5'→3') | Reverse (5'→3') | Product Size (bp) |
| --- | --- | --- | --- | --- |
| 1 | 626-645 | **ggctcacatcaccccataaa** |  | 2933 |
|  | 1230-1249 | caacctcaccacctcttgct |  |  |
|  | 1874-1893 | actttgcaaggagagccaaa |  |  |
|  | 2417-2436 | cactgtcaacccaacacagg |  |  |
|  | 3558-3539 |  | **tagaagagcgatggtgagag** |  |
| 2 | 3163-3182 | **gccttcccccgtaaatgata** |  | 2687 |
|  | 3662-3681 | cagggtgagcatcaaactca |  |  |
|  | 4233-4252 | tacaatctccagcattcccc |  |  |
|  | 4711-4730 | ccggacaatgaaccataacc |  |  |
|  | 5849-5830 |  | **caggggttaggcctcttttt** |  |
| 3 | 5451-5470 | **acactcatcgcccttaccac** |  | 2158 |
|  | 5849-5830 |  | caggggttaggcctcttttt |  |
|  | 6455-6436 |  | gaagaggggcgtttggtatt |  |
|  | 7038-7019 |  | agtggaagtgggctacaacg |  |
|  | 7608-7588 |  | **cctacttgcgctgcatgtgcc** |  |
| 4 | 7392-7410 | **ggatgccccccaccctacc** |  | 1760 |
|  | 8440-8421 |  | ttgggtgatgaggaatagtg |  |
|  | 8207-8226 | cccatcgtcctagaattaat |  |  |
|  | 9151-9132 |  | **ttaaggcgacagcgatttct** |  |
| 5 | 8855-8874 | **cgggcacagtgattataggc** |  | 2932 |
|  | 9438-9457 | ggccttcgatacgggataat |  |  |
|  | 10022-10048 | ccaattaactagttttgacaacattca |  |  |
|  | 10621-10640 | cccactccctcttagccaat |  |  |
|  | 11226-11245 | gctcccttcccctactcatc |  |  |
|  | 11786-11766 |  | **ttatgatgcgactgtgagtgc** |  |
| 6 | 11226-11245 | **gctcccttcccctactcatc** |  | 3631 |
|  | 12389-12370 |  | gggggaattagggaagtcag |  |
|  | 12984-12965 |  | gaggcctagtagtggggtga |  |
|  | 13645-13626 |  | agcgaggttgacctgttagg |  |
|  | 14270-14251 |  | ttcgggaggatcctattggt |  |
|  | 14856-14837 |  | **aggagtgagccgaagtttca** |  |
| 7 | 14619-14638 | **accccacaaaccccattact** |  | 2704 |
|  | 15195-15214 | tatccgccatcccatacatt |  |  |
|  | 15781-15801 | cccttttaccatcattggaca |  |  |
|  | 16413-16432 | tgaaatcaatatcccgcaca |  |  |
|  | 753-734 |  | **tgttccttttgatcgtggtg** |  |

Bold sequences denote primers using PCR. Sequences of the rest are used for internal sequence primers.
